# Visualization of Dialysis-Related Amyloid Arthropathy on ^18^F-FDG PET-CT Scan

**DOI:** 10.3390/diagnostics12010113

**Published:** 2022-01-05

**Authors:** Miju Cheon, Jang Yoo

**Affiliations:** Department of Nuclear Medicine, Veterans Health Service Medical Center, Seoul 05368, Korea; jang8214.yoo@gmail.com

**Keywords:** dialysis-related amyloidosis, amyloid arthropathy, long-term dialysis, FDG, PET-CT

## Abstract

We report a case of dialysis-related amyloid arthropathy in a patient with end-stage renal disease. It presented in our patient as moderately increased FDG uptake in the amyloid deposition in the periarticular tissues and eroding into adjacent bones.

A 76-year-old male patient with end-stage renal disease, secondary to polycystic kidney disease, presented with worsening swelling and pain in both shoulders and hips. He had been on regular hemodialysis for 20 years. On examination, there was pain, swelling and restriction of movements of the shoulder, wrist and hip. ^18^F-FDG PET-CT was performed to evaluate for renal mass observed during a regular follow-up for polycystic kidney disease. ^18^F-FDG PET-CT was performed using a standard PET-CT scanner (Discovery Molecular Imaging Digital Ready, GE Healthcare, Waukesha, WI, USA). The patient had fasted for six hours before scanning, and two sequential PET and CT scans were acquired at 60 min after ^18^F-FDG injection (175 MBq). The PET-CT demonstrated several hypermetabolic masses suggesting malignant lesions in both kidneys (Figure 1). There was also increased metabolic activity in thickened soft tissues surrounding hips (maximum SUV 5.52 on the left and 4.51 on the right), shoulders (Figure 2), left elbow and left wrist (Figure 3). A cystic collection along the right subscapularis and supraspinatus muscle was not hypermetabolic, but surrounded by a hypermetabolic rim. The associated non-contrast CT obtained in PET-CT imaging showed osseous erosions of both humeral and femoral heads.

Protein electrophoresis showed no monoclonal gammopathy. Serum kappa was 208.99 mg/L (normal 3.3–19.4 mg/L) and serum Lambda was 195.15 mg/L (5.71–26.3 mg/L) with a normal kappa-to-lambda ratio of 1.05 (normal 0.26–1.65). Serum β2-microglobulin was increased as 19.55 mg/L (normal 1.0–2.4 mg/L). Serum CRP and calcium were normal. Rheumatoid factor and anti-CCP antibodies were negative. No significant myocardial uptake was observed in the bone scan obtained after injection of ^99m^Tc-hydroxymethylene diphosphonate (HDP), which was performed to exclude rheumatoid arthritis due to arthralgia (Figure 4). Eventually, the patient underwent joint aspiration, and cytology revealed the presence of amyloidal deposits. Those findings were compatible with dialysis-related amyloid arthropathy.

Amyloidosis is characterized by the extracellular deposition of protein and protein derivatives. Dialysis-related amyloidosis (DRA) is a well-recognized and serious complication in patients on long-term dialysis. The duration of dialysis appeared to be a predominant risk factor, because amyloidosis occurred in patients with dialysis duration above 15 years [1]. DRA is characterized by the amyloid deposition with β2-microglobulin in the osteoarticular structure and viscera. Amyloid deposition with β2-microglobulin has a high affinity for collagen and predominantly affects the osteoarticular system [2]. Frequent sites of involvement are the shoulders, wrists, hips, knees and the carpal tunnel. Because the clinical manifestations of amyloid arthropathy can be easily confused with other polyarticular forms of arthritis such as rheumatoid arthritis, we should consider this diagnosis, particularly in patients with multiple myeloma or other predisposing conditions.

We report a case where the ^18^F-FDG PET-CT allows the identification of several DRA-associated lesions in the articular and periarticular soft tissues in a patient under long-term dialysis therapy. There were only a few reports dealing with ^18^F-FDG PET-CT findings on DRA [3,4]. It suggests that ^18^F-FDG PET-CT could be a non-invasive imaging modality showing the extent and distribution of osseous, articular and soft-tissue involvement in dialysis-related amyloid arthropathy.

## Figures and Tables

**Figure 1:**
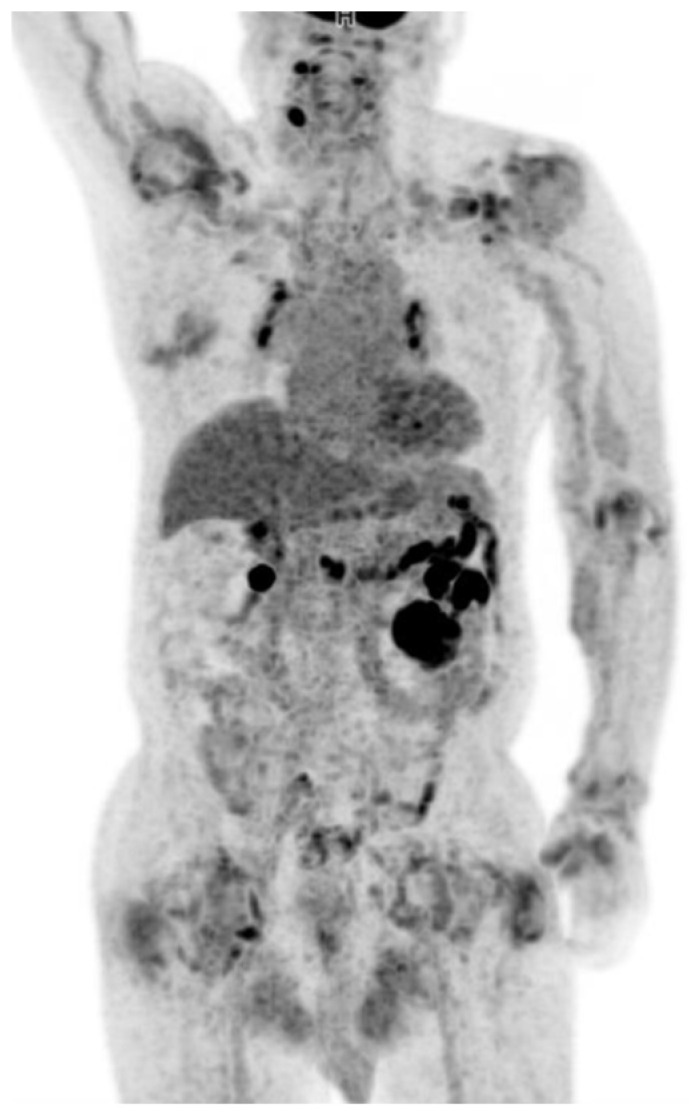
Maximum intensity projection of ^18^F-FDG PET-CT demonstrated increased metabolic activity in the shoulders, left elbow, left wrist, left hand and hips.

**Figure 2:**
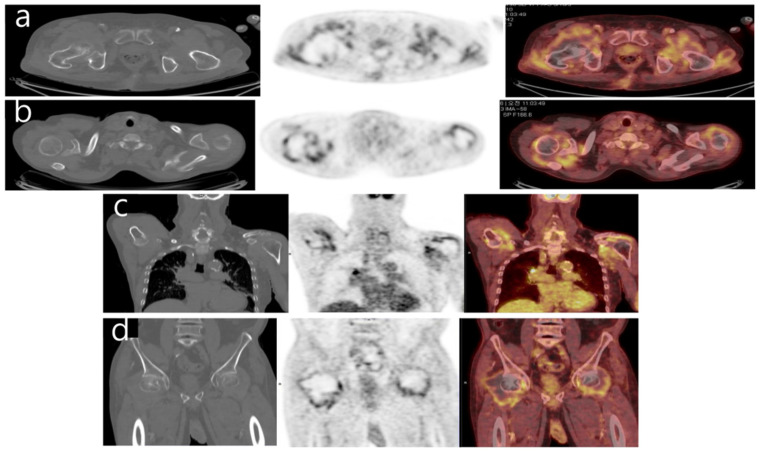
^18^F-FDG PET-CT axial (**a**,**b**) and coronal (**c**,**d**) images of both shoulders and hips demonstrate diffuse periarticular uptake. Non-contrast CT obtained in conjunction with PET-CT imaging showed osseous erosions involve both humeral and femoral heads.

**Figure 3:**
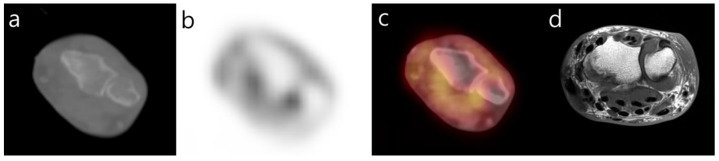
Axial ^18^F-FDG PET-CT image (**a**–**c**) of the left wrist demonstrates periarticular uptake and associated osseous erosions. Axial T1-weighted MR image (**d**) reconfirms erosion and periarticular hypointense amyloid deposits.

**Figure 4:**
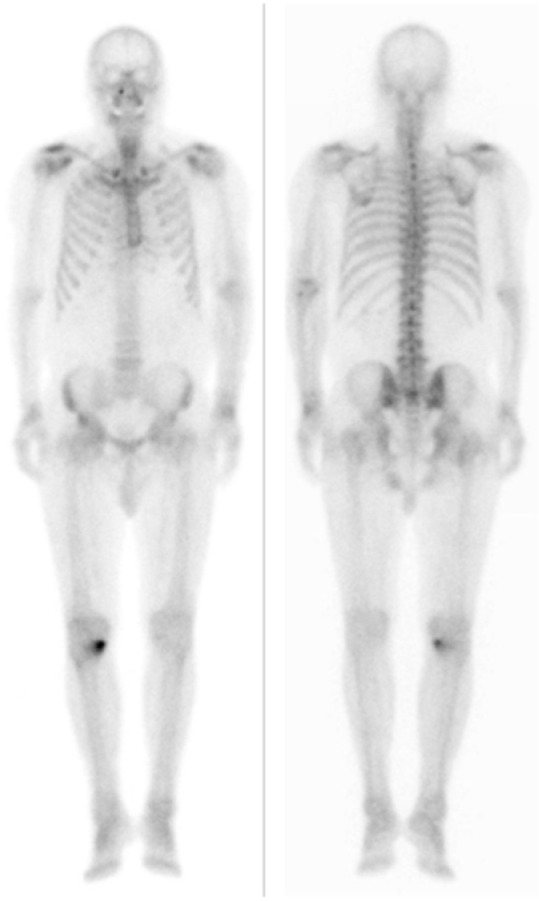
Bone scintigraphy with technetium-99m hydroxymethylene diphophonate (HDP) shows no cardiac accumulation.

## Data Availability

The data that support the findings of this study are available from the corresponding author M.C., upon reasonable request.
